# Sequential gene profiling of basal cell carcinomas treated with imiquimod in a placebo-controlled study defines the requirements for tissue rejection

**DOI:** 10.1186/gb-2007-8-1-r8

**Published:** 2007-01-15

**Authors:** Monica C Panelli, Mitchell E Stashower, Herbert B Slade, Kina Smith, Christopher Norwood, Andrea Abati, Patricia Fetsch, Armando Filie, Shelley-Ann Walters, Calvin Astry, Eleonora Aricó, Yingdong Zhao, Silvia Selleri, Ena Wang, Francesco M Marincola

**Affiliations:** 1 Immunogenetics Section, Department of Transfusion Medicine, Clinical Center National Institutes of Health, Bethesda, MD 20892, USA; 2 The Clinical Skin Center of Northern Virginia, Fairfax, VA 22033, USA; 3 3M Pharmaceuticals, St Paul, MN 55144-1000, USA; 4 Department of Dermatology, National Naval Medical Center, Bethesda, MD 20889, USA; 5 Laboratory of Pathology, National Cancer Institute, Bethesda, MD 20892, USA; 6 Biometric Research Branch, Division of Cancer Treatment and Diagnosis, National Cancer Institute, Bethesda, MD 20892, USA; 7 Universita' degli Studi di Milano, Department of Human Morphology, via Mangiagalli, 20133 Milan, Italy

## Abstract

**Background:**

Imiquimod is a Toll-like receptor-7 agonist capable of inducing complete clearance of basal cell carcinoma (BCC) and other cutaneous malignancies. We hypothesized that the characterization of the early transcriptional events induced by imiquimod may provide insights about immunological events preceding acute tissue and/or tumor rejection.

**Results:**

We report a paired analysis of adjacent punch biopsies obtained pre- and post-treatment from 36 patients with BCC subjected to local application of imiquimod (*n *= 22) or vehicle cream (*n *= 14) in a blinded, randomized protocol. Four treatments were assessed (q12 applications for 2 or 4 days, or q24 hours for 4 or 8 days). RNA was amplified and hybridized to 17.5 K cDNA arrays. All treatment schedules similarly affected the transcriptional profile of BCC; however, the q12 × 4 days regimen, associated with highest effectiveness, induced the most changes, with 637 genes unequivocally stimulated by imiquimod. A minority of transcripts (98 genes) confirmed previous reports of interferon-α involvement. The remaining 539 genes portrayed additional immunological functions predominantly involving the activation of cellular innate and adaptive immune-effector mechanisms. Importantly, these effector signatures recapitulate previous observations of tissue rejection in the context of cancer immunotherapy, acute allograft rejection and autoimmunity.

**Conclusion:**

This study, based on a powerful and reproducible model of cancer eradication by innate immune mechanisms, provides the first insights in humans into the early transcriptional events associated with immune rejection. This model is likely representative of constant immunological pathways through which innate and adaptive immune responses combine to induce tissue destruction.

## Abstract 2

An analysis of basal cell carcinoma subjected to local application of imiquimod revealed that most transcripts stimulated by imiquimod involve the activation of cellular innate and adaptive immune-effector mechanisms.

## Background

In 2004, Aldara™ (imiquimod 5% cream, 3M Pharmaceutical, St Paul, MN, USA) labeling was extended by the Food and Drug Administration to include treatment of superficial basal cell carcinoma (BCC) based upon randomized controlled trials demonstrating complete histological clearance in 78% to 87% of superficial BCC treated topically 5 days per week for 6 weeks [1,2]. Pilot-scale and investigator initiated trials had shown 90% to 100% clearance with q12 hours (twice per day) dosing [3].

Imiquimod belongs to a family of synthetic small nucleotide-like molecules with potent immuno-modulatory activity mediated through Toll-like receptor (TLR)-7 (and 8) signaling. When applied topically, these compounds display immune-mediated anti-tumoral activity without damaging normal tissues [1,3-7] Imiquimod targets predominantly TLR-7 expressing plasmacytoid dendritic cells (pDCs) with secondary recruitment and activation of other DC and macrophage subtypes and induction of T helper_1 _responses within three to five days of treatment [4]. Stimulation of pDCs through TLR-7/myeloid differentiation response gene 88 (My-D88)/IRF-7 signaling induces expression of interferon (IFN)-α, which appears to act upon natural killer (NK) cells and conventional dendritic cells (DCs) to stimulate IFN-γ, tumor necrosis factor (TNF)-α, monocyte chemoattractant proteins (MCPs) and other cytokines [5,8,9] This immunological cascade leads within two weeks to apoptotic death of cancer cells and their substitution by a mononuclear cell infiltrate [3-5,8]

Although imiquimod function seems particularly associated with IFN-α-stimulated genes (ISGs) [10], it remains unclear whether this pathway is solely responsible for all the downstream effects ultimately resulting in tumor clearance. Indeed, a comprehensive and conclusive characterization of the events leading to tumor rejection based on a prospectively controlled study has never been reported. We previously characterized ISGs *in vitro *[11] and *in vivo *(Belardelli F and Arico' E, manuscript in preparation), compiling a road map for the interpretation of transcriptional surveys of biological conditions affecting the tumor microenvironment (Additional data file 1).

Here, we report a paired analysis of adjacent punch biopsies obtained pre- and post-treatment from 36 patients with BCC subjected to local application of imiquimod or a control cream in a blinded, randomized protocol.

## Results

A total of 65 subjects were screened, but 27 were ineligible due to their pre-enrollment biopsy excluding BCC and 2 were ineligible for other reasons. A total of 36 subjects were eligible for the study and started treatment with either imiquimod (*n *= 22) or vehicle cream (*n *= 14) (Table 1). After unblinding, treatment groups were color-coded to facilitate the discussion. Out of the subjects, 61% had nodular BCC, 17% superficial BCC, and 22% unspecified BCC. Of note is that all 4 subjects randomized to the imiquimod q12 hours × 4 days group had nodular BCC. Post-treatment biopsies were taken <12 hours after last dose for 17% of subjects, >36 hours after the last dose date for another 17%, and between 18 and 30 hours after last dose for 33%. This variability was uncontrollable and due to patient compliance. The locations of the tumors were: 41% on the face; 25% on the extremities; 22% on the trunk; and 11% on either the neck or scalp. Furthermore, patient (P) 23 and P28 did not complete treatment, missing two placebo and one imiquimod dose, respectively. The imbalance in the distribution of the elapsed time between last treatment dose and post-treatment biopsy did not significantly affect the results except, possibly, for the q24 × 8 (pink) cohort. Interestingly, at this early time point, already 9 of 22 imiquimod-treated BCCs were found to be clear of tumor cells, particularly among patients treated with the most intense schedule.

### Quantitative PCR

**Figure 1:**
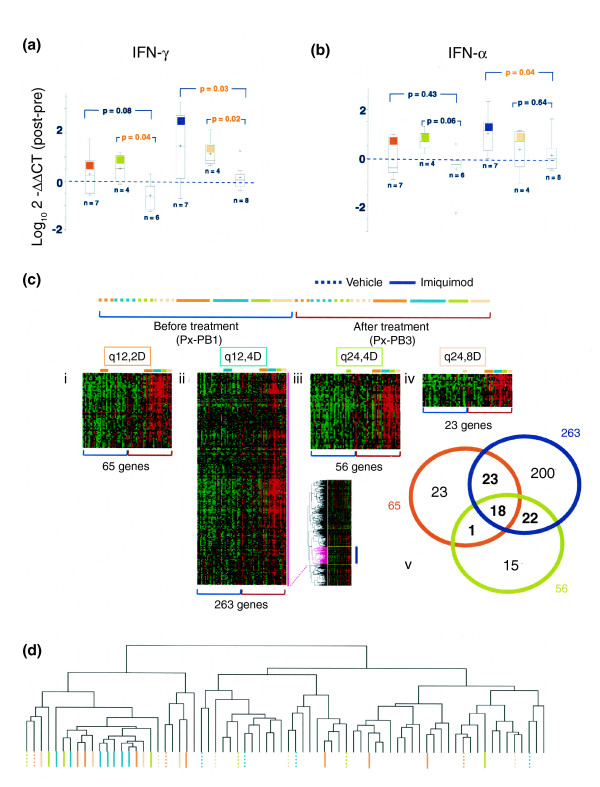
Differential expression of IFN-γ and IFN-α in EOT compared to pre-treatment samples in all cohorts; hierarchical clustering based on genes differentially expressed at EOT compared to pre-treatment samples in each treatment cohort and dendrogram showing the degree of relatedness of samples based on imiquimod-induced genes in the blue group. The 2^-ΔΔCT ^describes **(a) **IFN-γ and **(b) **IFN-α gene expression fold change at EOT relative to baseline after normalization according to the endogenous reference cyclophilin G. C_T _equals the mean cycle times of duplicate wells and ΔΔC_T _= (C_T_, Target-C_T_, cyclophilin) EOT - (C_T_, Target-C_T_, cyclophilin) baseline. The fold-change data were transformed using logarithm_10_. The box and whisker style box plot gives the median and interquartile range (box), 1.5 of the inter-quartile range (whiskers), points outside the whiskers (square symbols) and the mean (cross symbol). Statistics: *p *values refer to 2-sample *t*-tests between treatment and control groups. **(c) **Based on a paired *t*-test cut-off *p*_2 _value < 0.05, 1,311 genes were differentially expressed between the pre-treatment and EOT samples in the q12 × 2 (orange) cohort. Reclustering of these genes identified a node of 65 genes uniquely upregulated in the imiquimod-treated EOT samples (part i). Similar analyses were performed for the other imiquimod-treated cohorts; 1,578 genes were differentially expressed in the q12 × 4 (blue) cohort, including an imiquimod-specific node of 263 genes (part ii and the vertical blue bar in adjacent complete data set); 650 genes were differentially expressed in the q24 × 4 (green) cohort, including an imiquimod-specific node of 58 genes (part iii); and 495 genes were differentially expressed in the q24 × 8 (pink) cohort, including an imiquimod-specific node of 23 genes (part iv). A Venn diagram displays the extent of overlap among genes differentially expressed in the three most informative orange, blue and green groups (part v). **(d) **Reclustering of all BCC samples based on the imiquimod-specific 263-gene signature identified in the q12 × 4 (blue) cohort. Straight lines identify imiquimod-treated EOT samples color coded according to treatment regimen; dashed lines identify vehicle cream-treated EOT samples and unlabeled are the all pre-treatment samples. A diagram illustrating the strategy used to prepare Figure 1c,d is available as Additional data file 4.

At this early stage of treatment, no changes were observed in TNF-α and MCP-1 expression, in contrast with others' findings at later stages [5,8,9] IFN-γ 2^-ΔΔCT ^from baseline to end of treatment (EOT) was significantly increased compared to dose-matched controls at all but the earliest time point (q12 × 2, orange group; Figure 1a). IFN-α followed a similar pattern but significance was observed only with the most intense regimen (q12 × 4, blue group; Figure 1b).

### Identification of treatment (imiquimod)-specific genes

Unsupervised analysis applying various filtering parameters failed to segregate samples according to treatment, suggesting that imiquimod affects an insufficient number of genes to alter the global transcript of BCC. A paired *t*-test (cut-off *p*_2 _value < 0.05) was applied to identify genes differentially expressed by identical lesions before and after treatment within each cohort. For instance, the q12 × 4 (blue) cohort differentially expressed 1,578 genes at EOT compared to paired pre-treatment samples. Reclustering of these genes demonstrated that most were similarly expressed by post-treatment samples treated with placebo, reflecting changes due to vehicle alone or the tissue repair induced by the adjacent pre-treatment biopsy. A node, however, contained 263 genes exclusively upregulated in all EOT imiquimod-treated samples (Figure 1c (part b), vertical blue bar). This cohort-based training/prediction analysis was repeated with the other three treatment regimens, providing independently similar results. In all cases, nodes were identified inclusive of genes uniquely expressed in EOT imiquimod-treated samples (Figure 1c (parts a and d); Additional data file 4). The number of imiquimod-induced genes varied among cohorts, however, with the largest amount in the q12 × 4 (blue) cohort, in line with the higher clinical effectiveness of this intense dosing regimen [3]. There was extensive overlap among the genes identified by the various comparisons (Figure 1c (part e)); 41 (63%) of 65, 40 (71%) of 56 and 16 (70%) of 23 genes differentially expressed in the orange, green and pink groups, respectively, were included among those identified as differentially expressed in the blue group. Reclustering of experimental samples based on imiquimod-specific signatures from each cohort suggested their independent predictive value in sorting imiquimod-treated BCC from pre-treatment and control samples as exemplified by the blue cohort signature, which clumped together not only the samples from the blue group, which served as a basis to select the genes used for clustering, but also 9 of the other 15 imiquimod-treated samples compared with only 3 of 14 vehicle-treated samples (Fisher *p*_2 _value = 0.04). Four of the five samples that did not cluster together with the blue group samples belonged to the orange group (Figure 1d).

Thus, different dosing schedules differed quantitatively but not qualitatively, with the same genes being induced among them. The striking difference in number of genes induced between the q12 × 2 (orange) and the q12 × 4 (blue) cohorts strongly emphasizes the importance of the number of doses; however, the q24 × 8 (pink) group, which received the same number of imiquimod applications as the blue group in twice the amount of time, displayed similar but dampened transcriptional changes, emphasizing the importance of administration to sustain the pro-inflammatory stimulus associated with the higher efficacy of the q12 schedule.

**Figure 2:**
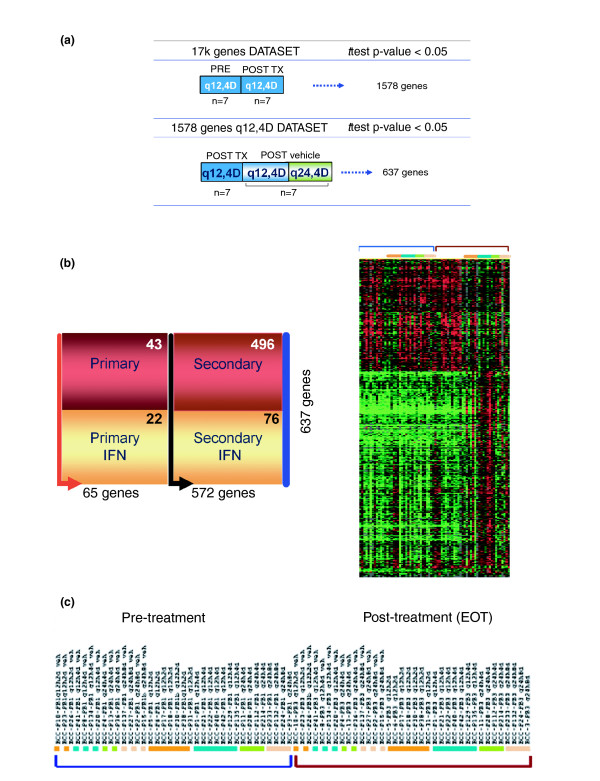
Identification of treatment (imiquimod)-specific transcripts in the most intensive schedule (q12 × 4 (q12,4d), blue cohort). **(a) **A pairwise *t*-test (*p *value < 0.05) was applied to identify genes differentially expressed between pre-treatment and EOT biopsies from the same BCC belonging to the q12 × 4 (blue) cohort. The 1,578 genes identified were then tested for treatment specificity by identifying those differentially expressed between the blue group treated with imiquimod (TX) compared with temporally matched, vehicle control-treated EOT biopsies (combined blue and green groups **(b) **The remaining 637 treatment-specific genes were classified based on their significant expression also in the earlier q12 × 2 (orange) group as primary (65 genes) while the other ones were considered secondary. Finally, the same genes were also compared to a database of IFN-α-associated transcripts as described in the Materials and methods. In the same panel the 637 genes are shown in a supervised-sample hierarchical clustering of the genes. **(c) **Legend of samples, dashed and solid bars identify vehicle control or imiquimod-treated samples, respectively.

This analysis supports the specificity of our findings but also simultaneously emphasized the need to discriminate imiquimod-specific effects from those due to vehicle cream application and/or tissue repair induced by the adjacent pre-treatment biopsy. Because q12 dose scheduling had been observed previously to produce the highest rates of clearance [3], we adopted this cohort as the basis for further analysis. This selection offered the additional advantage of allowing the largest number of temporally matched placebo-treated samples (q12 × 4 and q24 × 4 cohorts). At EOT, 1,578 genes were significantly altered in expression in the q12 × 4 (blue) cohort compared to pre-treatment (paired *t*-test cut-off *p *value < 0.05; Figure 2a). To eliminate placebo and/or surgical bias, an unpaired *t*-test (cutoff *p *value < 0.05) was applied to this gene pool, identifying transcripts differentially expressed between imiquimod-treated EOT samples and vehicle cream-treated samples. This analysis left 637 genes unequivocally modulated by imiquimod (Figure 2b,c; Additional data file 3). A global test was applied to this gene set to test the likelihood of getting this proportion of significant genes by chance (at the 0.05 level) if there were no real differences between the two classes. Such likelihood was negligible, with a permutation *p *value of 0.001. The false discovery rates (FDRs) of the differentially expressed genes are less than 11.9%. To estimate the specificity/accuracy of the 637 'imiquimod-induced' genes, we considered as a training set the samples utilized for their identification (q12 × 4 days treatment group and the q12 × 4 and q24 × 4 days vehicle groups; Figure 2b). The trained predictors were then used to segregate post-imiquimod treatment samples from pre-treatment or vehicle treated samples belonging to the other groups. This analysis was performed using the Support Vector Machines (a supervised learning algorithm that classifies data by finding optimal fit between different statistical classes); this analysis yielded a sensitivity of 60%, specificity of 92% and an overall accuracy of 82.4%. Thus, the set of 637 genes identified by this study represent a highly specific functional signature of imiquimod-induced changes during the early stages of therapy in lesions whose transcriptional profiles were sufficiently activated. The relatively low sensitivity of the gene set as predictors most likely reflects the exclusion of lesions in the earliest cohort (orange group) that were not exposed sufficiently to imiquimod.

Of the 637 genes, 65 were also significantly altered in expression in the q12 × 2 (orange) cohort; we refer, therefore, to these as 'primary' responders to imiquimod and to the rest as 'secondary'. Finally, the 637 genes were matched to our database of IFN-α-related signatures consisting of 426 genes identified using the same cDNA platform and reference system in monocytes stimulated with various IFN-α subtypes *in vitro *[11] and/or induced in *vivo *by systemic IFN-α_2b _therapy. Only 98 (22 included among the primary) genes matched the database and were considered *bona fide *ISGs. The primary ISGs included *STAT-1*, *MX1*, *MX2 *and *IFITM1*. By four days, secondary ISGs had broadened to *STAT2*, *IRF-2 *and *IRF7*, *JAK-2 *and *JAK-3 *and *N-myc interactor *(*NMI*). Moreover, CXCL10/IP-10 was significantly upregulated; CXCL10 is a monocyte and T lymphocyte chemoattractant interacting with the chemokine receptor CD183 (CXCR3) and T-cell CD26. The remaining 539 genes were induced through IFN-α-independent pathways, suggesting that only a small proportion of the effector activity of imiquimod is mediated by IFN-α.

#### Primary non-IFN-α-stimulated genes

**Figure 3:**
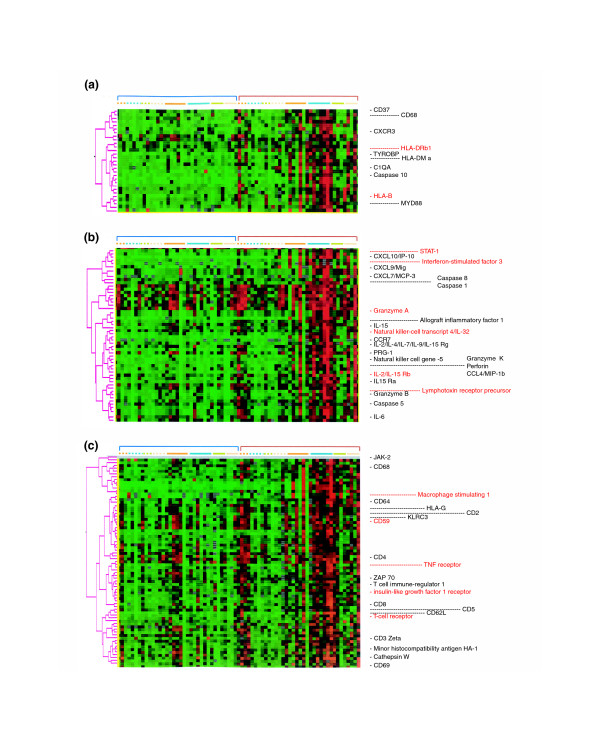
Visual display of selected treatment (imiquimod)-specific transcripts (complete database available on line). **(a) **Display of selected primary treatment-specific genes identified as per Figure 2. **(b) **Secondary treatment-specific genes related to effector functions with primary focus on cytokines, cytokine receptors and lytic enzymes. **(c) **Secondary treatment-specific genes representative of cell surface markers, receptors and associated molecules. In red are genes whose expression was found to be associated with acute renal allograft rejection [37]. Treatment cohorts are described by the bars on top of each cluster.

By the second day of q12 imiquimod treatment, 65 primary non-ISGs were identified, echoing predominantly innate immune effector functions (Figure 3a). CXCR3, a ligand for IP-10 and monokine induced by IFN-γ (MIG/CXCL9) was the earliest upregulated cytokine receptor, suggesting its early involvement in the crosstalk leading to migration and activation of monocytes and lymphocytes. Also induced by IFN-γ were several HLA class I and class II transcripts, including HLA-B and HLA-DRβ1. Transcripts critical for the activation of innate immune effector cells, such as NK cells and mononuclear phagocytes, were highly expressed; for example, TYROBP, a killer-cell immunoglobulin-like receptor family member and cytochrome β-245, a component of phagocytes' lytic function. Activation of macrophages was also strongly supported by the upregulation of CD68, and the modulation of complement component 1 qα (C1QA) and MY-D88 [12]. The induction of CD37 represented an early sign of the transition from an innate to an adaptive immune response as CD37 regulates T cell proliferation through TCR signaling [13]. Finally, Caspase 10 upregulation suggests an early initiation of apoptotic mechanisms.

#### Secondary non-IFN-α-stimulated genes

The vast majority of transcriptional effects were observed four days after q12 treatment (Figure 3b), when the inflammatory process is amplified by the induction of cytokines, their receptors and genes related to their interactions, such as dual specificity phosphatase 5 (DUSP-5) and the gene encoding the anti-apoptotic BCL2. The induction of pro-inflammatory molecules was strongly reminiscent of the broad transcriptional changes induced by the *in vitro *stimulation of peripheral blood mononuclear cells (PBMCs) by interleukin (IL)-2 [14]. In particular, the upregulation of cytokines and corresponding receptors within the common γ chain receptor family (particularly IL-15 and the IL-15 receptor α-chain, the IL-2/IL-15 receptor β-chain and the common γ chain itself; Figure 3b) suggest early activation within the tumor microenvironment of CD8 T and NK cells [15,16]. This notion is also supported by the modulation of downstream transcription factors of IL-2/IL-15 receptor triggering, such as Jak kinases, STAT-1, STAT-3 and STAT-5, and the upregulation of T cell receptor subunits, cytotoxic granules and NK-activation receptors (Figure 3b). The increased expression of the chemokine (C-C motif) receptor 7 (CCR-7) also supports a potent activation of pro-inflammatory signals; CCR7 is expressed by activated B and T lymphocytes and NK cells and controls their migration to inflamed tissues [17]. MIG is a chemoattractant for CXCR3-bearing immune cells that may contribute, together with IP-10, to the intensification of the acute inflammatory process. Monocyte inflammatory protein (MIP)-1α (CCL3), MIP-1β (CCL4) and MCP-3 (CCL7) were also induced at this point. Among them, MCP-3 has been shown to augment monocyte anti-tumor activity while CCL3/MIP-1α and MIP-1β represent potent pro-inflammatory factors with chemotactic properties for neutrophils and DC and NK cells. Interestingly, CD64 and the low-affinity IgG Fc receptor II-B (FCGR2B), which were also upregulated among the secondary non-ISGs (Figure 3c), have been shown to stimulate MIP-1α and MIP-1β release [18].

#### Cytotoxic T and NK cell signatures

The most striking effects of imiquimod were on cytotoxic mechanisms, with the induction of NK cell gene-5 (NKG-5), NK cell protein-4 (NK4)/IL-32 granzyme-B, -A and -K, perforin and lymphotoxin-β receptor [19,20]. (Figure 3b,c). Moreover, the concomitant transcription of several caspases indicate active cytotoxicity [20] combined with granule-mediated apoptosis suggested by the upregulation of proteoglycan 1 secretory granule (PRG1) [21].

Several T cell receptor signaling and amplification-associated genes were also upregulated, including those encoding TCR-α, -β and -γ chains, ζ-chain (ZAP70), CD3Z, T cell immune-regulator 1 and related co-receptor CD5 [22,23]. Moreover, CD2/LFA-2 mediates T and NK cell activation through interactions with CD59, which is also upregulated at this time point [24,25]. Similarly, the overexpression of CD69 marks the activation of T and NK cells and it has been correlated by Posselt *et al*. [26] with acute renal allograft rejection.

Several transcripts suggest a primary involvement of NK cells in the process, such as the NKG2 family of genes, which encode receptors that are expressed on most NK cells [27]: killer cell lectin-like receptor subfamily C, member 2 (KLRC2/NKG2C), member 3 (KLRC3/NKG2E), and member 4 (KLRC4/NKG2F). Moreover, all NK receptor adapter proteins containing an immune-receptor tyrosine based activation motif (ITAM) were found to be upregulated (FCERIg), CD3z and TYROBP/DAP12. The upregulation of KLRC2/NKG2C, TYROBP/DAP12 and FCER1G suggests the occurrence of NK and T cell activation, which would lead to release of pre-made cytotoxic granules and secretion of cytokines [27]. Another NK cell-related gene is that encoding Cathepsin w, a cysteine proteinase associated with the membrane and the endoplasmic reticulum of NK and T cells and regulation of their cytolytic activities [28]. Finally, the minor histocompatibility antigen HA-1 may be one of the immunodominant stimulators of graft-versus-host and graft-versus-malignancy effects through increasing cytotoxic mechanisms [29].

#### Markers of immune infiltrates

**Figure 4:**
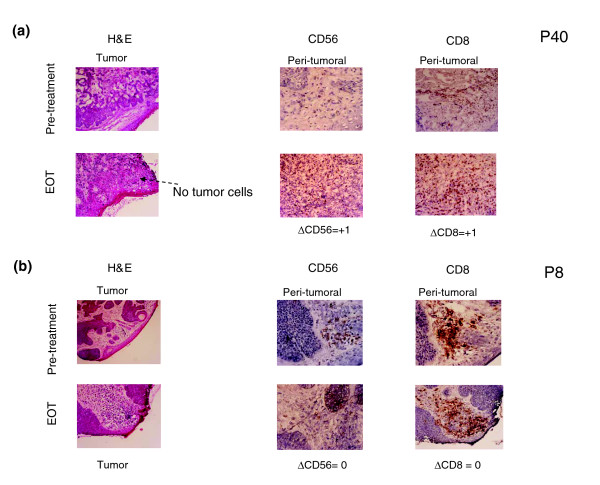
IHC staining for CD56 and CD8 in BCC from **(a) **P40 (imiquimod treated) and **(b) **P8 (vehicle-control). Lesions were graded blindly by two pathologists (AA and AF) and graded before and at EOT for peri-tumoral and intra-tumoral immune cells infiltrate. Cancer cells were evaluated separately for each marker. When BCC was absent at EOT as in P40 the immune infiltrate was compared to the peri-tumoral pre-treatment infiltrate. NE, not evaluable because no tumor cells were left at EOT.

Transcriptional analysis portrayed a predominant enhancement of immune infiltrates associated with T and NK cells. Because 9 of 22 imiquimod-treated BCCs were cleared of tumor cells at EOT it was impossible to further analyze whether the identified changes were occurring in specific histological areas as sharply defined in pre-treatment lesions. In such cases, changes in immune infiltrates were calculated comparing EOT results with pre-treatment peri-tumoral infiltrates. With all four imiquimod treatment groups pooled together, significant increases were noted in CD56 (NK cells), CD4 and CD8 T cells, with CD56 (NK cells) showing significant difference relative to the pooled vehicle group (Table 2, Figure 4). Moreover, BCL-2 expression was selectively enhanced in immune but not cancer cells. Importantly, enhancement of CD8 expression was strongly dependent upon treatment schedule, with 5 of 7 subjects treated in the q12 × 4 (blue) cohort experiencing increases in the number of CD8 T cells (*p *value < 0.05). Other markers did not reach statistical significance, including those associated with cytotoxic activity, such as granzymes and perforin, suggesting that the differences identified at the transcript level may precede changes detectable as protein expression, as we recently observed studying transcript to protein relationships in IL-2-stimulated PBMCs [14]. These data confirm the transcriptional observation that imiquimod primarily induces recruitment and activation of T and NK cells within the BCC microenvironment.

## Discussion

This is the first prospectively controlled study conducted to identify the early biological events associated with the eradication of BCC through an immune-mediated mechanism. By protocol design, tumor regression did not represent an endpoint and tumors were removed at the end of the study. Thus, the association between the molecular/genetic findings and tumor clearance is presumptive, based on the historical 80% to 90% clearance rates recognized by the Food and Drug Administration for the release of imiquimod for clinical use [2]. However, it is interesting to note that 9 of 22 (41%) imiquimod-treated BCCs were devoid of cancer cells by EOT (2 to 8 days from beginning of treatment) while only 1 of 14 (7%) control-treated BCCs had no identifiable tumor cells (Fisher test *p *value = 0.05), suggesting that artifacts due to vehicle administration or surgical trauma were not responsible for the early tumor clearance.

As indicated by qPCR, IFN-γ transcription was more prevalent than IFN-α transcription. This is in line with the evidence of predominant NK, CD8 and CD4 T cell activity in this study. Sullivan *et al*. [30] had indeed previously observed similar cellular infiltrates (particularly CD4 and CD56 expressing cells) in a smaller, open-label, matched controlled, non-randomized study in which six patients with BCC treated with imiquimod at daily intervals for a total of ten administrations were compared with six patients receiving comparable vehicle cream treatment. The predominance of IFN-γ transcription suggests that pDCs trigger other immune functions through the production of IFN-α, which in turn activates resident T and NK cells, selective producers of IFN-γ [31]. We hypothesize that these secondary immune effector mechanisms induce destruction of target cells, providing antigen to professional antigen presenting cells for priming of naive T-cells in draining lymph nodes [31,32]. Indeed, several of the transcripts associated with imiquimod treatment show activation of T and NK cells and induction of IFN-γ stimulated genes (Figure 3). The cytotoxic T and NK cell signatures identified here (granzymes, perforin and other NK cell-related genes) have recently been described in a mouse model of IFN-α and IFN-γ-producing killer DCs (IKDCs) [33], which simultaneously display cytotoxic and pro-inflammatory functions. Thus, IKDCs could summarize in a cellular unit our findings of ISG activation combined with broader cytotoxic and pro-inflammatory properties. At present, IKDCs have not been characterized in humans, nor it is known whether they express TLR-7; future studies should address their role as putative mediators of immune rejection.

Imiquimod treatment stands as a unique opportunity to study the mechanisms of immune-mediated rejection directly in human tissues. This TLR-7 agonist links multiple immune pathways. Of these, IFN-α plays a consistent but not exclusive role. Previous transcriptional surveys have provided a broad view of the biological processes associated with immune-mediated tissue destruction, identifying convergent characteristics. Neoplastic inflammation approaches the unresolving process of chronic hepatitis C virus (HCV) infection where the presence of antigen-specific immune responses do not lead to clearance of the pathogen in the majority of cases [34,35]. Both diseases are characterized by the expression of ISGs that do not seem sufficient to clear the pathogenic procress. Similar signatures can be identified in liver biopsies from patients with chronic HCV infection [36] and in chronic allograft rejection controlled with standard immune suppression [37]. ISGs are also consistently expressed in melanoma metastases following the systemic administration of IL-2 independent of clinical outcome [38]. Thus, it appears that ISGs are part of immunological processes associated with chronic inflammation insufficient to clear its cause. On the contrary, several non-ISGs identified by this study delineate potent inflammatory (CCL7/MCP-3, CCL4/MIP-β, and so on) and cytotoxic (granzymes, perforin, NKG-5, and so on) functions rarely observed in chronically inflamed tissues but described in the acute inflammation associated with destruction of a tumor [38] or allograft [37], liver damage in HCV-induced cirrhosis [36] or gut dysfunction during flares of Crohn's disease [39]. This study corroborates the impression that immune-mediated tissue destruction comprises at least two components: a baseline cluster of ISGs that may be necessary but insufficient to induce tissue rejection and a less common activation of broad cytotoxic and other potent pro-inflammatory innate immune effector functions that are more tightly associated with rejection. Our findings are supported by the recent description of clearance of established cancers by the adoptive transfer of innate immune effector cells in the powerful model of spontaneous regression/complete resistance mice [40].

Sarwal M *et al*. [37] reported strikingly similar results evaluating the transcriptional behavior of renal cell allograft during acute rejection, basing the analysis on a similar array platform and utilizing the same RNA amplification method [41] (Figure 3, transcripts labeled in red). In spite of these similarities, they also reported a B cell signature characterized by enhanced expression of CD20 and several immunoglobulins that we did not identify in our study. This discrepancy could be explained by a specific role that B cell-mediated immunity may play in the context of allo-recognition. In the case of BCC, the strong pro-inflammatory stimulus induced by imiquimod through TRL-7 signaling might bypass the requirement for an endogenous, tissue specific insult responsible for the secondary triggering of the cellular immune effector mechanisms identified by both studies. The signatures identified by both studies also match the anecdotal identification of the same genes in a melanoma metastasis that underwent regression following systemic IL-2 therapy [38].

Among the genes mutually reported by the previous three studies, NK4/IL-32 was recently recognized as a central mediator of Crohn's disease [42] and associated with liver damage during HCV infection [36]. NK4/IL-32 is a potent inducer of pro-inflammatory cytokines and it is selectively expressed by immune cells stimulated with IFN-γ IL-2 or the combination of IL-12 and IL-18 [14,39]. Indeed, we found NK4/IL-32, together with other genes associated with cytotoxic function, to be constitutively expressed by NK cells but only by activated CD8+ T cells [43]. Moreover, we recently observed NK4/IL-32 to be preferentially expressed in metastatic melanoma compared with other less immune responsive cancers [44]. It is possible that NK4/IL-32 may play a central role during imiquimod treatment by amplifying inflammatory stimuli through the induction of a cytokine cascade. Thus, this novel cytokine emerges as a central player in immune rejection or autoimmunity.

Dermatologists have long used imiquimod to treat BCC [4,45,46] Imiquimod mimics the action of single-stranded viral RNA [31], activating a pro-inflammatory cascade as a chemical prototype of the danger model of immune activation [47]. Meanwhile, tumor immunologists have struggled to explain the paradoxical co-existence of tumor antigen-specific T cells induced by vaccination with growing tumor tissues. Indirect evidence suggests that vaccine-induced T cells reach the tumor site [48] and recognize tumor cells producing IFN-γ˜
 MathType@MTEF@5@5@+=feaafiart1ev1aaatCvAUfeBSjuyZL2yd9gzLbvyNv2Caerbhv2BYDwAHbqedmvETj2BSbqee0evGueE0jxyaibaiKI8=vI8tuQ8FMI8Gi=hEeeu0xXdbba9frFj0=OqFfea0dXdd9vqai=hGuQ8kuc9pgc9s8qqaq=dirpe0xb9q8qiLsFr0=vr0=vr0dc8meaabaqaciGacaGaaeqabaqadeqadaaakeaaiiaacuWFZoWzgaacaaaa@34E8@ However, this is not sufficient for tumor rejection since other effector mechanisms are not simultaneously activated [49] because cancers do not provide the danger signal necessary for full implementation of the immune responses [50]. Thus, immunization successfully affects the afferent loop of the immune response by eliciting TA-specific T cells but cannot affect T cell activation at the receiving end [51,52]. The cancer specificity of TLR agonists consists of the preferential attraction of TLR-7 expressing pDCs to chronically inflamed tissues and their enhanced recruitment [53]. Similar conclusions were recently reached by Torres *et al*. [54], who followed the biological events induced by imiquimod when administered to patients with actinic keratosis. Thus, TLR agonists exemplify how the gap between the induction of TA-specific T cells by immunization and their activation at the receiving end could be closed. It is thus conceivable that preparations of TLR agonists suitable for systemic administration may be used in the future as single agent therapy for other tumor types (trials are currently ongoing in Europe for melanoma) or as adjuvants to enhance the effectiveness of active-specific immunization approaches [55-57].

## Conclusion

This study stands as a proof of principle that, when tissues are easily accessible, mechanistic observation about the effects of a treatment can be easily performed in humans by combining minimally invasive techniques (fine needle aspirates, through cut or punch biopsies) with high-fidelity mRNA amplification; such approaches are fundamental to refresh scientific hypotheses through direct human observation. Second, it provides insights into the early events leading to tumor rejection in a most powerful human model. Finally, it suggests that immune-mediated tumor rejection is only one aspect of tissue-specific destruction, which follows a constant immunological pathway shared by other anti-cancer immunotherapies, acute allograft rejection, autoimmune disease and tissue damage during chronic pathogen infections.

## Materials and methods

Detailed methods are available as Additional data file 2.

### Study design and patient information

This double-blind, placebo-controlled, randomized, parallel group clinical trial sponsored by 3M Pharmaceuticals and registered before patient enrollment (3M/NNMC study #1454-IMIQ) was designed to evaluate the early transcriptional events induced by topical imiquimod administration. The trial was conducted at the National Naval Medical Center (Bethesda, MD, USA) in compliance with the Code of Federal Regulations and the guidelines for Good Clinical Practice. Imiquimod (5%, 12.5 mg) or vehicle cream were supplied in single-use 250 mg sachets. Following biopsy confirmation and time for healing, subjects applied a sufficient quantity of cream to cover the entire BCC and an area approximately 2 cm around. Each dose was left on the skin for eight hours. For the study, 48 subjects were supposed to be randomized in a 2:1 ratio to either imiquimod or vehicle within each of 4 dosing regimens (q12 hours for 2 or 4 days or q24 hours for 4 or 8 days). Subjects were randomized at the time of screening when the pre-enrollment biopsy was taken. Once eligibility was determined based on the biopsy result, the investigator contacted the subject, who either started treatment on a date instructed by the investigator or returned the study drug. Replacement subjects were identified for all subjects with a biopsy result negative for BCC or who discontinued prior to EOT procedures. BCCs were to be a least 7 mm diameter and were to be located on the scalp, face, trunk or proximal extremities. Punch biopsies (PB; 2 mm diameter) were obtained pre-enrollment to verify the diagnosis of BCC, pre-treatment (PB1 and PB2) and at EOT (PB3 and PB4), approximately 24 hours after the last dose taken. PB1 and PB3 were transferred immediately at the bedside into cryovials with 2 μl Rnalater (Ambion, Austin, TX, USA), frozen in liquid nitrogen and stored at -80°C for total RNA isolation. PB2 and PB4 were placed in a cryomold, filled with OCT compound (Tissue-Tek, Elkhart, IN, USA), frozen in liquid nitrogen and stored at -80°C for immunohistochemistry (IHC).

### RNA isolation and amplification and cDNA arrays

Total RNA was isolated with RNeasy minikits (Qiagen, Germantown, MD, USA) and amplified into anti-sense RNA as previously described [41,58,59] with the following modifications to minimize RNA degradation by abundant skin RNAases. Samples were homogenized in disposable tissue grinders (Fisher Scientific, Lafayette, CO, USA). Proteins potentially interfering with RNA isolation were removed by incubating the homogenate in 590 μl distilled water and 10 μl PROTEINASE K solution (Qiagen) at 55°C for 10 minutes then centrifuged at ambient temperature for 3 minutes. Supernatants were combined with 0.5 volumes of ethanol (96% to 100%) into a Rnase-Dnase free tube and RNA was isolated through a RNeasy mini column. First strand cDNA synthesis was accomplished in 1 μl SUPERase•In (Ambion) and ThermoScript RT (Gibco BRL, Gaithersburg, MD, USA) in 2 μg bovine serum albumin. RNA quality was verified by Agilent technologies (Palo Alto, CA, USA). Anti-sense RNA was used for probe preparation or quantitative real-time PCR (qPCR). For microarray analysis, test samples were labeled with Cy5-dUTP (Amersham, Piscataway, NJ, USA) and co-hybridized with reference pooled normal donor PBMCs labeled with Cy3-dUTP to custom made 7 K-cDNA microarrays [60]. Arrays were scanned on a GenePix 4000 (Axon Instruments, Union City, CA, USA) and analyzed using Cluster and Tree View software [61]. Gene ratios are presented according to the central method for display [62]. Gene annotations were mined using web-based tools such as DAVID [63], GeneCards [64], COPE [65] and Bioinformatic Harvester [66].

### Quantitative PCR

QPCR was applied to detect the expression of IFN-α, IFN-γ, TNF-α and MCP-1 using an ABI Prism 7900 HT (Applied Biosystems, Foster City, CA, USA). Primers and probes were custom-designed to span exon-intron junctions and generate <150 base-pair amplicons (Biosource, Camarillo, CA, USA). Taqman probes were labeled at the 5' and 3' ends with the reporter FAM (6-carboxyfluorescein; emission λ_max _= 518 nm) and the quencher TAMRA (6-carboxytetramethylrhodamine; emission λ_max _= 582 nm), respectively. Standard curves were based on amplicons generated from human leukocyte antigen (HLA)-A*0201 expressing lymphocytes stimulated with IL-2 (300 IU/ml) and Flu M1:58-66 peptide; copy numbers were estimated with Oligo Calculator [67]. Linear regression R^2^-values pertinent to all standard curves were ≥ 0.98. QPCR reactions were conducted in a 20 μl volume, including 1 μl cDNA, 1× Taqman Master MIX (Applied Biosystems), 2 μl of 20 μM primer and 1 μl of 12.5 μM probe. Thermal cycler parameters included 2 minutes at 50°C, 10 minutes at 95°C and 40 cycles involving denaturation at 95°C for 15 s, annealing-extension at 60°C for 1 minute. The 2^-ΔΔCT ^method was utilized to compute fold change in gene expression at EOT relative to baseline after normalization according to cyclophilin G expression [68].

### Immunohistochemistry

After confirming the presence of epidermis, dermis and tumor using hematoxylin and eosin, IHC was performed by staining 7 mm consecutive acetone-fixed sections for the expression of CD4, CD8, CD56, CD95, FasL, granzyme A and B, perforin, BCL-2, TRAIL, caspase 3 and PARP. Secondary staining consisted of biotinylated goat-anti-mouse IgG followed by avidin-biotin-peroxidase. A semi-quantitative estimation was conducted to separate histological entities as: tumor cells; intra-tumoral immune infiltrate; and peri-tumoral immune infiltrate. Scoring was assigned independently by two blinded pathologists (AA and AF) as: 0 (none), 1+ (few), 2+ (moderate), 3+ (numerous). Data are presented as shift in scores at EOT compared to baseline.

### Statistics

Significance testing was based on paired or 2-sample two-tailed Student *t*-test as appropriate. *P *values < 0.05 were considered statistically significant. No adjustment was made for multiple comparisons. Fisher exact test was used to test the level of significance comparing the frequency of events between treatment groups. All analyses related to class comparison and class prediction was done using the BRB-Array Tools [69] developed by Simon [70]. Microarray raw data were curated according to GEO (series # GSE5121) [71].

## Additional data files

The following additional data are available with the online version of this paper. Additional data file 1 provides a list of genes previously shown to be associated with the stimulation of various cell types with IFN-α. Additional data file 2 is an extended version of the Materials and methods, providing full disclosure of the methodology used. Additional data file 3 provides a complete list of the 637 genes specifically induced by imiquimod treatment based on the statistical approach presented in the text. Additional data file 4 is a diagram illustrating the mining strategy that was implemented for the preparation of Figure 1c,d.

## Supplementary Material

Additional data file 1 List of genes previously shown to be associated with the stimulation of various cell types with IFN-α. Click here for file

Additional data file 2 Extended version of the Materials and methods, providing full disclosure of the methodology used. Click here for file

Additional data file 3 Complete list of the 637 genes specifically induced by imiquimod treatment based on the statistical approach presented in the text. Click here for file

Additional data file 4 The mining strategy that was implemented for the preparation of Figure 1c,d. Click here for file

## Figures and Tables

**Table 1:** Composition of study cohorts

Patient ID	Cohort	Doses received	EOT → B× time lapse (hours)	Histology	ΔCD8	ΔCD56	Tumor at EOT
P5	Imiq q12 × 2 days	4	13	Nodular	0	-1	+
P6	Imiq q12 × 2 days	4	14	Undetermined	0	0	+
P17	Imiq q12 × 2 days	4	36	Undetermined	NE	NE	-
P18	Imiq q12 × 2 days	4	33	Nodular	+1	0	+
P30	Imiq q12 × 2 days	4	16	Nodular	0	0	-
P38	Imiq q12 × 2 days	4	17	Nodular	0	0	+
P231	Imiq q12 × 2 days	4	22	Undetermined	+1	0	+
P10	Vehic q12 × 2 days	4	12	Nodular	0	0	+
P23	Vehic q12 × 2 days	2	15	Nodular	+2	0	+
P26	Vehic q12 × 2 days	4	45	Nodular	0	0	+
			Mean ± SD = 22 ± 11.5				
							
P1	Imiq q12 × 4 days	8	8	Nodular	0	0	+
P21	Imiq q12 × 4 days	8	41	Nodular	0	+1	+
P22	Imiq q12 × 4 days	8	11	Nodular	+1	0	-
P40	Imiq q12 × 4 days	8	17	Nodular	+1	+1	-
P42	Imiq q12 × 4 days	8	3	Undetermined	+3	0	-
P129	Imiq q12 × 4 days	8	19	Nodular	+1	0	+
P135	Imiq q12 × 4 days	8	21	Superficial	+1	+1	-
P41	Vehic q12 × 4 days	8	28	Nodular	+1	0	+
P134	Vehic q12 × 4 days	8	19	Nodular	+2	0	+
P8	Vehic q12 × 4 days	8	20	Nodular	0	0	+
P20	Vehic q12 × 4 days	8	16	Superficial	0	0	+
			Mean ± SD = 18 ± 10.2				
							
P11	Imiq q24 × 4 days	4	26	Nodular	0	+1	+
P28	Imiq q24 × 4 days	3	20	Nodular	NE	NE	+
P112	Imiq q24 × 4 days	4	44	Nodular	0	0	+
P214	Imiq q24 × 4 days	4	51	Nodular	+2	+1	-
P4	Vehic q24 × 4 days	4	16	Superficial	NE	-1	-
P13	Vehic q24 × 4 days	4	30	Nodular	0	0	+
P36	Vehic q24 × 4 days	4	25	Superficial	0	0	+
			Mean ± SD = 30 ± 12.8				
							
P233	Imiq q24 × 8 days	8	32	Undetermined	0	+1	+
P132	Imiq q24 × 8 days	8	159	Undetermined	0	+2	-
P24	Imiq q24 × 8 days	8	48	Superficial	+1	0	-
P3	Imiq q24 × 8 days	8	12	Undetermined	-1	0	+
P2	Vehic q24 × 8 days	8	6	Undetermined	0	-1	+
P15	Vehic q24 × 8 days	8	21	Nodular	0	0	+
P27	Vehic q24 × 8 days	8	26	Nodular	NE	NE	+
P137	Vehic q24 × 8 days	8	11	Superficial	0	0	+
			Mean ± SD = 39 ± 50.3				

Punch biopsies are labeled according to patient number (P1 to P42) and timing of excision: PB0, pre-enrollment; PB1 and PB2, pre-treatment; PB3 and PB4, post-treatment. Biopsies from patients replacing drop-outs were labeled one digit to the serial number (that is, P101 to P142 or P201 to P242. PB1 and PB3 were collected for total RNA isolation; PB2 and PB4 for IHC. Undetermined refers to a BCC histology in-between superficial and nodular. ΔCD8 and ΔCD56 scores differences in infiltrate between EOT and pre-treatment samples (see Materials and methods). Tumor at EOT: identifiable (+) or not identifiable (-) tumor cells in the hematoxylin eosin stained EOT biopsy. Imiq, imiquimod; NE, not evaluated; Vehic, vehicle.

**Table 2:** Scoring of immune infiltrate by immuno histochemistry

Pooled treatment groups	Δ Score post - pre-treatment							Within group p value
		
	-3	-2	-1	0	1	2	3	
CD56								
Imiquimod	0	0	1	12	6	1	0	0.03
Vehicle	0	0	2	11	0	0	0	0.17
CD8								
Imiquimod	0	0	1	10	7	1	1	0.01
Vehicle	0	0	0	9	1	2	0	0.22
CD4								
Imiquimod	0	0	1	12	7	0	0	0.03
Vehicle	0	0	2	5	4	1	0	0.22
BCL-2								
Imiquimod	0	0	1	11	6	2	0	0.02
Vehicle	0	0	2	9	1	1	0	0.72

*P *values associated with the paired *t*-test for within group shifts relative to baseline. Δ Score refers to differences in infiltrate between EOT and pre-treatment samples using the scoring scale described in Materials and methods (IHC section).
