# Supplementary material for: Sequential gene profiling of basal cell carcinomas treated with imiquimod in a placebo-controlled study defines the requirements for tissue rejection
Source: Genome Biol. 2007 Jan 15;8(1):R8. doi: 10.1186/gb-2007-8-1-r8 (PMC1839129; doi:10.1186/gb-2007-8-1-r8)
Supplement: Additional data file 2 — Extended version of the Materials and methods, providing full disclosure of the methodology used. [file gb-2007-8-1-r8-S2.doc]

**Sequential gene profiling of basal cell carcinomas treated with imiquimod in a placebo-controlled study defines the requirements for tissue rejection.**

Monica C. Panelli^1^, Mitchell E. Stashower^2^, Herbert B. Slade^3^, Kina Smith^1^, Christopher Norwood^4^, Andrea Abati^5^, Patricia Fetsch^5^, Armando Filie^5^, Shelley-Ann Walters^3^, Calvin Astry^3^, Eleonora Arico^1^, Yingdong Zhao^6^, Ena Wang^1^ and Francesco M. Marincola^1^

**Affiliation:**

^1^Immunogenetics Section, Department of Transfusion Medicine, Clinical Center National Institutes of Health, Bethesda MD, 20892, ^2^The Clinical Skin Center of Northern Virginia, Fairfax, VA, 22033, ^3^3M Pharmaceuticals, St. Paul, MN, 55144-1000, ^4^Department of Dermatology, National Naval Medical Center, Bethesda, MD 20889, ^5^Laboratory of Pathology, National Cancer Institute, Bethesda, MD 20892, ^6^Biometric Research Branch, Division of Cancer Treatment and Diagnosis, National Cancer Institute, Bethesda, MD 20892.

**Running title:** Molecular profiling of Basal Cell carcinoma treated with Imiquimod

*Address correspondence to Dr. Francesco M. Marincola at Department of Transfusion Medicine, Clinical Center, Building 10, Room 1C-711, 10 Center Drive MSC 1502, Bethesda, MD 20892-1502, U.S.A.

[Fmarincola@mail.cc.nih.gov](mailto:Fmarincola@mail.cc.nih.gov)

**DETAILED METHODS**

***Ethical Conduct of the Study***

This clinical trial was conducted in compliance with the Code of Federal Regulations (CFR) of the United States (US) Food and Drug Administration (FDA) (21 CFR Part 56, Institution Review Boards, and 21 CFR Part 50, Protection of Human Subjects) and ICH E6, Guideline for Good Clinical Practice.

***Study design and patient accrual***

This study was designed as a double blind, placebo controlled, randomized, parallel group clinical protocol registered before patient enrollment (3M/NNMC study #1454-IMIQ; National Institutes of Health Clinical Center (CC) Protocol# 02-CC-0289,Clinical trial # NCT00045851) and was sponsored by 3M Pharmaceuticals (3M), St. Paul, MN. Imiquimod/ Aldara^TM^ or matching vehicle cream was supplied in a single-use sachet containing 250mg of cream. Each sachet contained 12.5 mg of Imiquimod. Subjects were randomized to one of 4 treatments regimens as defined in **Table 1**. The maximum possible exposure to Imiquimod was either 50 mg (4 doses) or 100 mg (8 doses).

Following biopsy confirmation and time for healing, subjects applied a sufficient quantity of cream to cover the entire BCC and an area approximately 2 cm around. Each dose was left on the skin for 8 hrs. Forty-eight subjects were to have been randomized in a 2:1 ratio to either imiquimod or vehicle within each of 4 dosing regimens (q12hours for 2 or 4 days or q24 hours for 4 or 8 days). Subjects were randomized at the time of screening when the pre-enrollment biopsy was taken. Once eligibility was determined based on the biopsy result, the investigator contacted the subject who either started treatment on a date instructed by the investigator or returned the study drug. Replacement subjects were identified for all subjects with a biopsy result negative for BCC or who discontinued prior to end or treatment (EOT) procedures.

***Sample collection and freezing***

BCC were to be a least 7 mm diameter and were to be located on the scalp, face, trunk or proximal extremities. Punch biopsies (2 mm diameter) were obtained pre-enrollment to verify the diagnosis of BCC, pre-treatment (PB1and PB2) and at EOT (PB3 and PB4), approximately 24 hours after the last dose taken. PB1 and PB3 were, transferred immediately at the bedside into cryovials with 2 μl Rnalater (Ambion, Austin, TX). The vials were capped and immersed in a Dewar flask containing 200-500 ml of liquid nitrogen and flesh frozen for 1-2 min then transferred to -80C freezer for storage for subsequent total RNA isolation. PB2 and PB4 were collected for immunohistochemistry (IHC) analysis and immediately placed after resection in a cryomold, filled with O.C.T. compound (Tissue-Tek, Elkhart, IN), frozen in liquid nitrogen and stored at -80^o^C for immunohistochemistry (IHC) then flesh frozen in liquid nitrogen similarly to the samples intended for RNA isolation. Alternatively the biopsy was placed in OCT medium on a microscope slide incubated on dry ice. Cryomolds and OCT blocks made on slides were sealed in plastic bags and stored at 80 C.

***RNA isolation from skin samples and modified RNA amplification for microarrays and Taqman real time PCR***

Total RNA was isolated using RNeasy minikits (Qiagen, Germantown, MD). Amplified antisense RNA (aRNA) was prepared from total RNA (0.5-3 μg) according to a modified version (see modification to original protocol, A-C below) of the protocol previously described by us (1;2;2). The modifications introduced for total RNA isolation were the following: A) Tissue was homogenized through a 15ml disposable small tissue grinder (Fisher Scientific cat# 06-434A) containing originally 150μl of lysis buffer (RLT buffer + β-ME) and subsequently an additional 200 μl of lysis buffer to complete tissue homogenization and lysis. The lysate was then transferred to a QIAshreddder spin column and centrifuged at for 2 minutes at max speed.

B) To remove proteins that interfered with RNA isolation, 590μl double-distilled water were added to the homogenate followed by 10μl PROTEINASE K solution (Qiagen) and mixed thoroughly by pipetting. The homogenate was then incubated at 55°C for 10 minutes and centrifuged for 3 min at 13000 rpm at room temp. The supernatant (approximately 900μl) was then combined and mixed well with 0.5 volumes (usually 450 μl) of ethanol (96-100%) into a new Rnase-Dnase free tube mixed well by pipetting. To Separate RNA from DNA. 700μl of the sample (including any precipitate that may have formed) were transferred into a RNeasy mini column placed in a 2ml collection tube. Total RNA was isolated according to the manufacturer protocol. The modifications introduced for Antisense RNA amplification were the following:

C) First strand cDNA synthesis was accomplished in the presence of 1ul SUPERase•In (Ambion Cat# 2696 stored at –20 °C) instead of Rnasein Ambion Cat# 2696 stored at –20 °C) and ThermoScript RT (Gibco BRL Cat# 12236-022) instead of Superscript . One microliter of BSA (2ug/ul) was added as a carrier to each samples to increase RNA yield. D) A higher concentration of 1^st^ round of amplification material was achieved by resuspending antisense RNA into ½ (11 ul) of original procedure volume and taking all of 1^st^ round amplification-material to the second round of amplification. Quality of RNA was tested by Agilent technologies and spectophotometry (OD). Antisense RNA was either used for probe preparation and microarray analysis or for Taqman real time PCR. For microarray analysis test samples were labeled with Cy5-dUTP (Amersham, Piscataway, NJ) while the reference sample (pooled normal donor PBMC) was labeled with Cy3-dUTP. Test-reference sample pairs were mixed and co-hybridized to 17K-cDNA microarrays generated in our lab (gal file <http://nciarray.nci.nih.gov/gal_files/gal_custom_current.shtml>, [Hs-CCDTM17.5k-1px.gal](http://nciarray.nci.nih.gov/gal_files/Hs-CCDTM17.5k-1px.gal)).

***Microarray analysis***

Hybridized arrays were scanned at 10-μm resolution on a GenePix 4000 scanner (Axon Instruments, Union City, CA) at variable PMT voltage to obtain maximal signal intensities with < 1% probe saturation. Resulting tiff images were further analyzed using Cluster and Tree View software (3) and Partek Pro software (Partek Inc., St Charles, MO). The global gene expression profiling data set of pre and post treatment specimens included 72 experimental samples. For unsupervised analysis, low stringency filtering was applied in the Eisen’s clauster program (3) (90% gene presence across all experiments and removal of genes that did not have at least in 1 of the samples a Log _2_ ≥ 2; 4 ratio) which reduced the original data base to 6646 genes. Supervised clustering of experimental samples was performed either by directly applying different filters on the global data set or by reducing the number of genes by statistical analysis (Student’s paired and unpaired 2 tail t-Test). Gene ratios were average corrected across experimental samples and displayed according to the central method for display using a normalization factor as recommended by (4)

***Microarray mining analysis***

Mining of microarray data was performed using the following web based mining tools:

[**http://david.abcc.ncifcrf.gov/**](http://david.abcc.ncifcrf.gov/)**,**

[**http://www.genecards.org/index.shtml**](http://www.genecards.org/index.shtml)

[**http://www.copewithcytokines.de**](http://www.copewithcytokines.de/)

**Bioinformatic harvester/Stanford University** [**http://harvester.embl.de/harvester/P599/P59901.htm**](http://harvester.embl.de/harvester/P599/P59901.htm)

***Taqman real time PCR***

Taqman real time PCR was performed on amplified RNA material isolated from punch biopsies in conditions identical to those applied for cDNA arrays and was applied to detect the expression of IFN-α, IFN-γ, TNF-α, MCP-1 transcripts. ABI Prism 7900 HT Sequence Detection System with 384well capability (Applied Biosystem, Foster City, CA) was utilized for detection. Primers and Taqman probes (Biosource, Camarillo, CA) were designed to span exon-intron junctions and to generate amplicons of <150bp. Taqman probes were labeled at the 5’ end with the reporter dye molecule FAM (6-carboxyfluorescein; emission λ_max_=518nm) and at the 3’ end with the quencher dye molecule TAMRA (6-carboxytetramethylrhodamine; emission λ_max_ =582nm). The following are the sequences for forward (f) and reverse (r) primer and probe (p) pairs

Sequences for Primers/probe sets -Taqman PCR.

TNF A#3 f GGAGAAGGGTGACCGACTCA

TNF A#3 r TGCCCAGACTCGGCAAAG

TNF A#3 p 6-FAM – CGCTGAGATCAATCGGCCCGACTA -TAMRA

IFNA1#2 f TTCCTCCTGTCTGATGGACAGA

IFNA1#2 r TGGAACTGGTTGCCATCAAA

IFNA1#2 p 6-FAM – TGACTTTGGATTTCCCCAGGAG-TAMRA

IFNG #2 f CCAACGCAAAGCAATACATGA

IFNG #2 r TTTTCGCTTCCCTGTTTTAGCT

IFNG #2 p 6-FAM – TCATCCAAGTGATGGCTGAACTGTCGC -TAMRA

MCP1 #2 f CATGGTACTAGTGTTTTTTAGATACAGAGACTT

MCP1 #2 r TAATGATTCTTGCAAAGACCCTCAA

MCP1 #2 p 6-FAM – AACCACAGTTCTACCCCTGGGATG-TAMRA

Cyclophilin G #2 f TGAGCATGATCACAGTAAAAGTAAGGA

Cyclophilin G #2 r TTGTTCTGCTATTATAACTGTGTTTACCTTTAG

Cyclophilin G #2 p6-FAM – AAGGATAGACGCGCACAATCCAGGAGT-TAMRA

Standards for the selected genes were amplified by reverse transcriptase primer specific amplification of 6 μg anti-sense-RNA obtained from PBMC stimulated in vitro with IL-2 (300 IU/ml and Flu M1 peptide) and reverse transcribed using random dN_6_ primers (Boehringer Mannheim, Germany). Amplified cDNA standards were quantified by spectrometry and number of copies was calculated using the Oligo Calculator software program ([http://micro.nwfsc.noaa.gov/protocols/oligoTMcalc.html).](http://micro.nwfsc.noaa.gov/protocols/oligoTMcalc.html).Standard)

Six microgram of test anti-sense RNA samples were converted to cDNA using random primers and immediately used for quantitative real time PCR (RT-PCR). RT–PCR reactions of cDNA samples were conducted in a total volume of 20 μl including 1 μl cDNA, 1x Taqman Master MIX (Applied Biosystems), 2 μl of 20 μM primers and 1ul of 12.5 μM probe. Thermal cycler parameters included 2 min at 50 ºC, 10 min 95ºC and 40 cycles involving denaturation at 95ºC for 15 sec, annealing-extension at 60 ºC for 1 min.

***Taqman real time PCR analysis***

Two different methods were used to analyze transcript changes in IFN-alpha, IFN-gamma, TNF-alpha and MCP-1 induced by Imiquimod treatment: 1) relative quantification (copy number), based on standard curves of the genes of interest and 2) fold changes based on the2–ΔΔCT method.

Linear regression analyses of all standard curves were ≥ 0.98. Standard curve extrapolation of copy number and quantity means were performed using the ABI Prism SDS 2.1 software (Applied Biosystem). Normalization of samples was performed by dividing the quantity mean of the gene of interest run in duplicate by the quantity mean of reference cyclophilin g gene x 10^5^.

The 2–ΔΔCT method was utilized to compute the fold change in gene expression at End of treatment (EOT, PB3) relative to baseline (PB1), normalized to the endogenous reference gene Cyclophilin G according to the method by Livak and Schmittgen (5). CT is the mean cycle times of the duplicate well readings, and ΔΔCT = (CT, Target - CT, Cyclophilin) EOT - (CT, Target - CT, Cyclophilin) Baseline.

The fold-change data were transformed using logarithm-base-10 (log_10_). Within group shifts in the transformed data relative to baseline were examined with the paired t-test. The 2-sample T-tests were used to compare each active Imiquimod group to the corresponding pooled vehicle group (with respect to the transformed data). In addition, the log_10_-transformed data were ranked and an ANOVA test was performed to compare the rank means of the Imiquimod groups with the corresponding vehicle groups.

Similar analyses were performed on the fold-change data computed using the absolute copy number. In this method, fold-change was computed using the relative ratio of absolute copy numbers at (EOT) relative to baseline, normalized using Cyclophilin data. The following formula was used: (EOT Cytokine/EOT Cyclophilin) / (Baseline Cytokine/Baseline Cyclophilin).

***Immunohistochemistry***

Hematoxylin and Eosin (H&E) staining was performed on all lesions to identify the presence of epidermis, dermis and tumor prior to immunohistochemistry staining.

We stained consecutive sections of the same lesions for the expression of the markers displayed below.

| **Stain** | **Manufacturer** | **Titer** | **Fixative** | **Pattern** | **Control** |
| --- | --- | --- | --- | --- | --- |
| CD4 | Becton-Dickinson | 1:20 | Acetone | Membrane | TIL1520 |
| CD8 | Becton-Dickinson | 1:20 | Acetone | Membrane | TIL 1520 |
| CD56 | BioGenex | 1:50 | Acetone | Membrane | TIL1520 |
| Fas (CD95) | Novocastra | 1:20 | Acetone | Membrane | TIL1520 |
| FasL | Novocastra | 1:20 | Acetone | membrane/cytoplasmic | TIL1520 |
| Granz A | BD PharMingen | 1:100 | Acetone | granular cytoplasmic | TIL1520 |
| Granz B | Novocastra | 1:20 | Acetone | granular cytoplasmic | TIL1520 |
| Perforin* | Novocastra | 1:10 | Formalin | granular cytoplasmic | TIL1520 |
| Bcl-2 | Zymed | 1:1 | Acetone | cytoplasmic | Jurkat |
| TRAIL | Alexis(nuclear pattern)  Novocastra (cytoplasmic pattern) | 1:10 | Acetone | membrane/cytoplasmic | anti Fas treated Hela |
| Caspase3 active | Pharmingen | 1:200 | Acetone | cytoplasmic | anti Fas treated Hela |
| PARP | Promega |  | Acetone | nuclear | Anti-Fas-Jurkat |

The staining with purified monoclonal antibodies was performed on 7um frozen section preparations of BCC that were fixed in acetone or formalin. Appropriate positive and negative controls were evaluated (see *Preparation of control for apoptosis markers*). For secondary staining, biotinylated goat-anti-mouse IgG (Kirkegaard & Perry Laboratories, Gaithersburg, MD) was used followed by avidin-biotin-peroxidase (Vector Laboratories, Burlingame, CA). Three different histological areas were analyzed as described in the next section: 1) tumor, 2) lymphocytes outside the tumor and 3) lymphocytes infiltrating the tumor.

***Immunohistochemistry Analysis***

A semi-quantitative classification was conducted on each section. Lymphocytes inside and outside the tumor were scored as: 0 (none), 1+ (few), 2+ (moderate), 3+ (numerous). Within-group shifts in scores at EOT relative to baseline were examined with the paired t-test.

Tumor was scored in a semi-quantitative fashion with percent of immunoreactive tumor cells (<25%, 25-<50%, 50-<75%, and >75%) and intensity of stain (1+, 2+, and 3+). The composite scoring used was as follows: 0 (negative), 1 (<25% 1+), 2 (25-<50% 1+ or <25% 2+), 3 (50-75% 1+ or <25% 3+), 4 (>75% 1+ or 25-<50% 2+) 6(25-<50% 3+ or 50-75% 2+), 8 (>75% 2+), 9 (50-75% 3+), and 12 (>75% 3+). Within-group shifts in composite scores at EOT relative to baseline were examined with the paired t-test.

***Preparation of control for apoptosis markers***

Jurkat and Hela cells treated with anti-fas (Pharmingen, San Diego, CA) or camptotecin (Sigma, St.Louis, MO) were used as positive control for apoptosis stainings by IHC. Cytospins were obtained from Jurkat cells treated with anti-Fas 5.4ul (100ng/ml) for 4 hours at the concentration of 1x10^6^ per well of a 24 well plate. HL-60 cells were plated at 2x10^6^ cells in 2ml of RPMI and treated with 1ul of camptothecin (5ug/ml) for 3 hrs or with 5.4ul of anti-Fas (100ng/ml) for 4 hrs. TIL 1520 (an archival T cell line kindly provided by Dr. Steven Rosenberg, Surgery Branch, NCI/NIH) activated with IL-2 (6000 IU/ml) was used as positive control for lymphocytes markers.

**References**

(1) Wang E, Miller L, Ohnmacht GA, Liu E, Marincola FM. High fidelity mRNA amplification for gene profiling using cDNA microarrays. Nature Biotech 2000; 17(4):457-459.

(2) Wang E, Marincola FM. Amplification of small quantities of mRNA for transcript analysis. In: Bowtell D, Sambrook J, editors. DNA arrays - A Molecular Cloning Manual. Cold Springs Harbor, NY: Cold Spring Harbor Laboratory Press, 2002: 204-213.

(3) Eisen MB, Spellman PT, Brown PO, Botstein D. Cluster analysis and display of genome-wide expression patterns. Proc Natl Acad Sci U S A 1998; 95(25):14863-14868.

(4) Ross DT, Scherf U, Eisen MB, Perou CM, Rees CA, Spellman PT et al. Systematic variation in gene expression patterns in human cancer cell lines. Nature Genetics 2000; 24(3):227-235.

(5) Livak KJ, Schmittgen TD. Analysis of relative gene expression data using real-time quantitative PCR and the 2(-Delta Delta C(T)) Method. Methods 2001; 25(4):402-408.
