# Supplementary material for: Sequential gene profiling of basal cell carcinomas treated with imiquimod in a placebo-controlled study defines the requirements for tissue rejection
Source: Genome Biol. 2007 Jan 15;8(1):R8. doi: 10.1186/gb-2007-8-1-r8 (PMC1839129; doi:10.1186/gb-2007-8-1-r8)
Supplement: Additional data file 4 — The mining strategy that was implemented for the preparation of Figure 1c,d. [file gb-2007-8-1-r8-S4.ppt]

## Slide 1
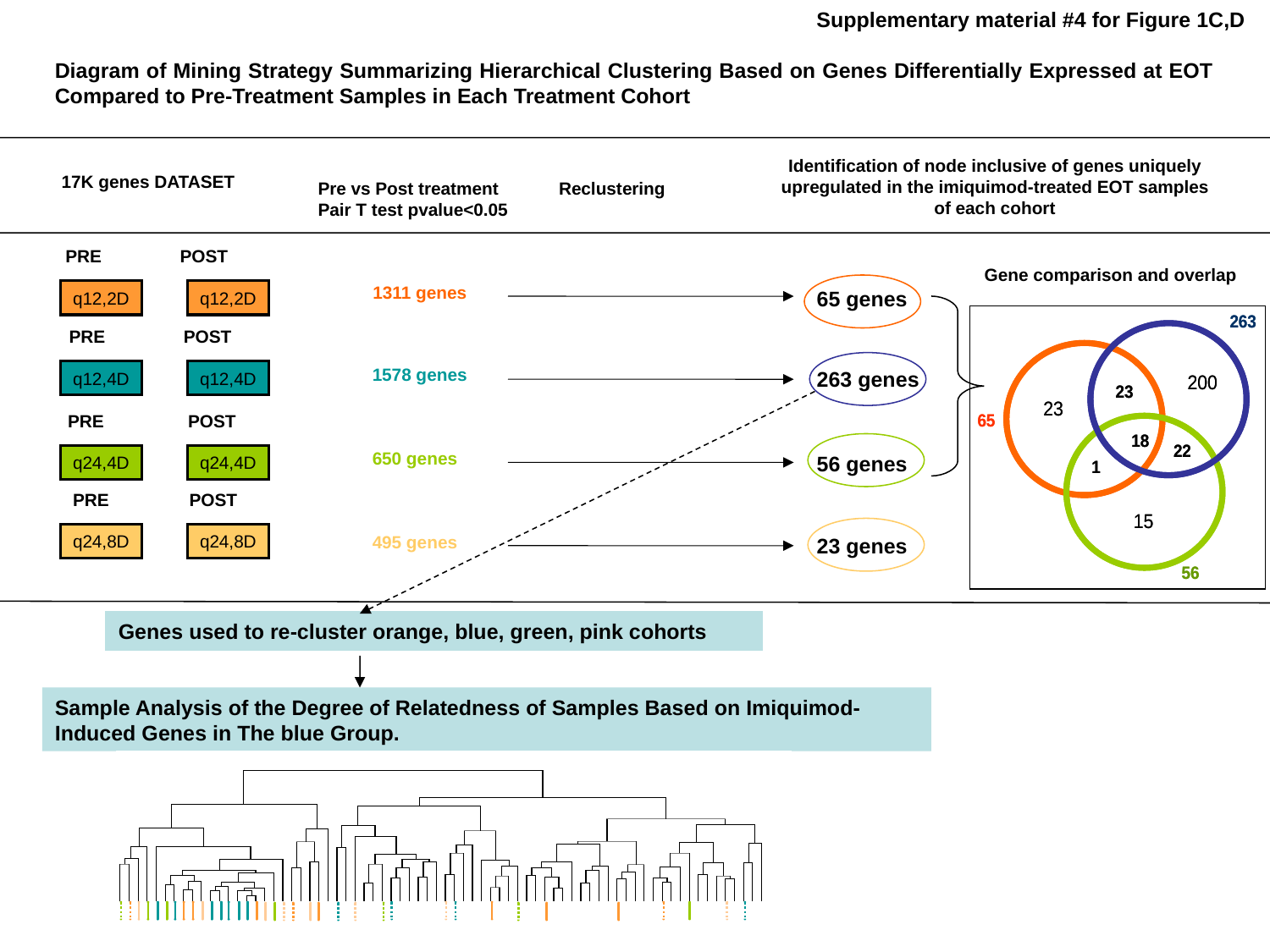

Supplementary material #4 for Figure 1C,D
Diagram of Mining Strategy Summarizing Hierarchical Clustering Based on Genes Differentially Expressed at EOT Compared to Pre-Treatment Samples in Each Treatment Cohort
Identification of node inclusive of genes uniquely upregulated in the imiquimod-treated EOT samples of each cohort
17K genes DATASET
Pre vs Post treatment
Pair T test pvalue<0.05
Reclustering
PRE
POST
q12,2D
q12,2D
Gene comparison and overlap
1311 genes
65 genes
PRE
POST
q12,4D
q12,4D
1578 genes
263 genes
PRE
POST
q24,4D
q24,4D
650 genes
56 genes
PRE
POST
q24,8D
q24,8D
495 genes
23 genes
Genes used to re-cluster orange, blue, green, pink cohorts
Sample Analysis of the Degree of Relatedness of Samples Based on Imiquimod-Induced Genes in The blue Group.
